# *How do my distributions differ?* significance testing for the overlapping index using permutation tests

**DOI:** 10.3758/s13423-026-02946-z

**Published:** 2026-07-13

**Authors:** Giulia Calignano, Ambra Perugini, Massimo Nucci, Livio Finos, Massimiliano Pastore

**Affiliations:** 1 Department of Developmental and Social Psychology, University of Padova, Padova, Italy; 2 Department of General Psychology, University of Padova, Padova, Italy; 3 Human Inspired Technology Research Centre, University of Padova, Padova, Italy; 4 Department of Statistical Sciences, University of Padova, Padova, Italy; 5 Padova Neuroscience Center, University of Padova, Padova, Italy

**Keywords:** Simulation, Type I error, Data visualization, Reaction time, Nonparametric inference

## Abstract

Psychological research frequently relies on statistical tests targeting single distributional parameters, typically means, despite empirical data often differing in variance, skewness, or overall shape. We introduce the $\zeta _{\textrm{ov}}$ test, a permutation-based inferential procedure built on the Overlapping Index, an effect size quantifying similarity between empirical distributions. The proposed approach evaluates global distributional differences without relying on parametric assumptions. Through simulations manipulating mean, variance, skewness, and sample size, we examine the $\zeta _{\textrm{ov}}$ test alongside commonly used tests (t, Welch, Wilcoxon–Mann–Whitney, Kolmogorov–Smirnov, and variance tests), while acknowledging that these tests address different null hypotheses. Results indicate that the $\zeta _{\textrm{ov}}$ test maintains adequate Type I error control under the simulated scenarios and shows comparatively high sensitivity to distributional differences, particularly when these involve more than a single parameter. An applied example using reaction-time data shows how distributional overlap detects differences missed by mean-based analyses. Rather than replacing traditional tests, the method provides a theoretically aligned global assessment that encourages distribution-aware inference and integration of visualization and descriptive analysis into statistical workflows. The $\zeta _{\textrm{ov}}$ framework supports ongoing methodological shifts in the psychological sciences toward robust, assumption-light, and interpretable statistical reasoning.

## Statistical testing choices in psychology

Cognitive psychology frequently relies on comparisons between experimental conditions to infer psychological effects. Standard analyses typically focus on single summary statistics, such as mean differences, and depend on assumptions that are rarely satisfied by behavioral data. Yet cognitive measures often differ in dispersion, skewness, or tail behavior rather than central tendency alone. Researchers also often need to demonstrate that groups are comparable before interpreting experimental effects, for instance, to argue that observed differences are attributable to a manipulation rather than pre-existing sample characteristics. In practice, this comparability is usually inferred from non-significant differences in group means, sometimes accompanied by separate tests of variance, implicitly treating equality of means as evidence of overall similarity. However, groups with similar averages may still differ substantially in distributional structure, leading researchers to rely on multiple parameter-specific tests that provide only a partial assessment of similarity. In this work, we propose a distribution-based statistical test that evaluates both similarity and difference by measuring the overlap between empirical distributions. The test derives from the Overlapping Index (Gini & Livada, 1943; Pastore et al., 2024), which is the area intersected by two or more probability density functions, making it possible to quantify the similarity or difference among samples (Inman & Bradley, 1989). The implementation of the test through permutations allows global differences to be tested without relying on rigid assumptions.

The need for a distribution-level approach is especially clear in research areas where psychological data naturally depart from symmetry and homogeneity assumptions. Reaction time paradigms, such as attentional cueing, e.g., the Posner cueing task, Calignano et al. (2024); Posner (1980), lexical decision tasks (Balota & Yap, 2011; Gastaldon & Calignano, 2025), social cueing tasks (Geiger et al., 2018; Lorenzoni et al., 2023), and eye-tracking or pupillometric studies (Calignano et al., 2021; Cavanagh et al., 2014), typically yield right-skewed distributions containing outliers (Heathcote et al., 2002; Ratcliff, 1993). In these contexts, mean-based tests may indicate no difference even when the distributions differ substantially in spread or shape. Nevertheless, even significant mean effects would fail to capture the full structure of the data (Matzke & Wagenmakers, 2009; Pastore & Calcagnì, 2019; Rieger & Miller, 2020).

Comparable patterns emerge in other areas of psychological science. Developmental studies often reveal changes in variability and behavioral strategies rather than average performance (Byers-Heinlein et al., 2021; ManyBabies Consortium, 2020; Rouder & Haaf, 2019). Similarly, in clinical contexts, comparisons of anxiety scores between clinical and control groups, or between patients and caregivers (Spaggiari et al., 2024), may involve similar mean scores alongside pronounced differences in extreme responses (Knowles & Olatunji, 2021). Across these domains, the inferential question shifts from whether averages differ to whether distributions themselves differ, motivating methods that directly quantify distributional similarity e.g., overlapping indices (Inman & Bradley, 1989; Pastore & Calcagnì, 2019).

Awareness of the data-generating process is increasing, along with concern about the uncritical use of statistical tools and analytical methods without adequate consideration of their assumptions and implications. Scheel et al. (2021) caution against the routine and uncritical application of hypothesis tests detached from theoretical grounding, advocating for theory-aligned analysis. In this spirit, the present proposal not only aims to guide the researcher in an aware and thoughtful choice of statistical tests, but also advocates for the implementation of descriptive statistics and data visualization in the routine workflow of data analysis, encouraging a more explicit focus on the structure of empirical distributions. As originally noted by Fisher (1925), the choice of a statistical test should always be guided by the context and purpose of its application.

This contribution introduces a novel approach to statistical testing, particularly for comparing two groups or conditions. Specifically, the paper proposes a new test, called the $\zeta _{\textrm{ov}}$ test, which applies the Permutation Test (Pesarin, 2001) alongside the Overlapping Index (η, Pastore and Calcagnì, 2019) to compare empirical distributions. The Overlapping Index and the proposed test aim to promote reflection on the structure and shape of the data rather than uncritical use of a new inferential tool. The test should not be used as a mere alternative test with fewer assumptions, but as a deliberate choice in targeted cases (i.e. preliminary checks between groups/conditions, cases of violation of assumptions).

The $\zeta _{\textrm{ov}}$ test has four main advantages. First, the core strength of the $\zeta _{\textrm{ov}}$ test is that it derives from an effect size and as such is highly intuitive. By quantifying the degree of overlap between two density distributions, it supports a straightforward interpretation of the test Perugini et al. (2026). The focus of the test on the entire distribution of the data, combined with the recommendation to include descriptive statistics and data visualization in analytical workflows, contributes to increasing awareness about the generative process of the data, as already mentioned, essential for drawing meaningful conclusions in psychological research.

The second advantage is its ability to simultaneously account for and compare the mean, variance, and shape with a single test. The $\zeta _{\textrm{ov}}$ test is a global distributional test, and may reduce the inflation of false positive rates that can arise when multiple parameter-specific tests are applied sequentially, a scenario commonly encountered in preliminary or baseline checks. This particular use of the test aligns with Scheel et al. (2021) recommendations by avoiding fishing expeditions for significant effects.

The third advantage lies in the fact that the proposed test can also be naturally extended to paired samples, making it suitable for repeated-measures designs commonly adopted in experimental psychology. In these settings, differences between conditions are evaluated within the same individuals, allowing individual variability to be explicitly preserved. The permutation procedure is implemented by shuffling condition labels within participants rather than across groups, thus maintaining the dependency structure of the data. This approach incorporates individual differences directly into the inferential process while providing a distribution-level comparison between conditions. An example based on real data illustrating this application is presented in the article, and all code required to reproduce the analyses is openly available.

Lastly, the $\zeta _{\textrm{ov}}$ test offers an alternative tool to address the assumptions issue. Given that parametric tests commonly used for statistical inference rely on strong assumptions, such as normality and homoscedasticity, assumptions which are unlikely to be met in many areas of psychological research (e.g., reaction times, accuracy, proportions), alternative methods like this one may be useful. In cases of small sample sizes, when tests are more sensitive to assumption violations, an alternative choice that is more adherent to the empirical distribution of the data is preferable.

**Fig. 1:**
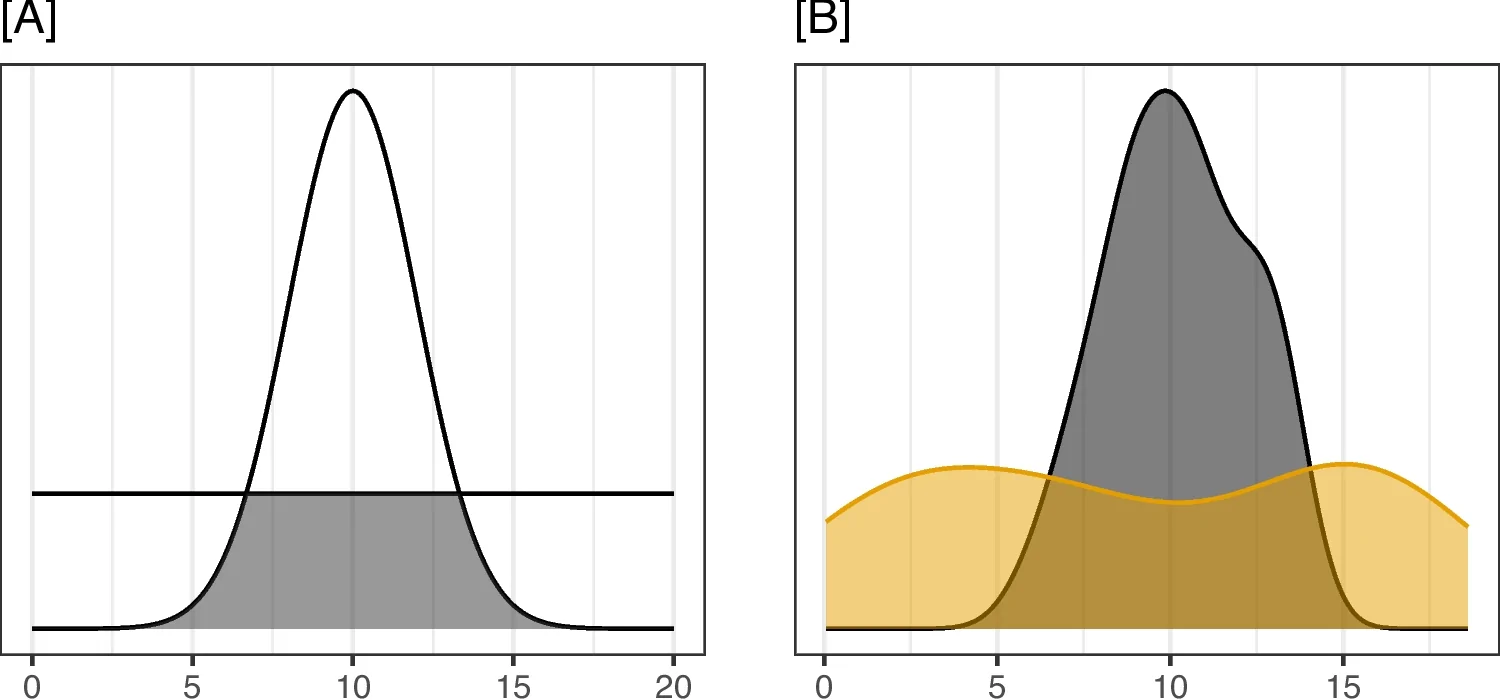
Comparison of a normal distribution and a uniform distribution with same mean, 10, and different variances, 4 and 33.3, respectively

The remainder of this article is structured as follows. First, we introduce the concept of the Overlapping Index, providing foundational definitions and highlighting its importance. Next, we define the Permutation approach and explore its application to the Overlapping Index. With a practical application on a real case of RTs, evidence is provided for its relevance in statistical analysis. Subsequently, we present a Simulation study to illustrate the practical implications and the performance of the Overlapping Index utilizing permutations. By simulating different scenarios, we compare statistical tests used in the psychological sciences with this novel approach, evaluating their control of Type I error and power. Finally, we discuss the results and provide the readers with an easy-to-implement workflow using the $\zeta _{\textrm{ov}}$ test, offering insight into the strengths and limitations of the Permutation-based Overlapping Index and its potential applications in psychological sciences.

## Methods

### Overlapping index

The Overlapping Index (η) is an intuitive way to define the area intersected by two or more empirical density functions (Pastore & Calcagnì, 2019). In a simple way, two distributions are similar when their distribution functions overlap, and as η diminishes, the two distributions differ. The η index varies from zero – when the distributions are completely disjoint – to one – when they are completely overlapped (Pastore, 2018). The simple interpretation of the overlapping index (η) makes its use particularly suitable for many applications (e.g. Jensen & Sanner, 2021; Garofalo et al., 2022; Schuetze & Yan, 2023; Karrobi et al., 2023; Sirbiladze et al., 2024; Habibi et al., 2024; Ricote et al., 2024; Rossi et al., 2024; Einbeck et al., 2024; Wachendörfer & Oeberst, 2024; Rohrbach, 2024; Hawkins et al., 2024; Upadhayay et al., 2024; Conversano et al., 2024; Pietrabissa et al., 2024; Nougaret et al., 2024; Greene et al., 2024)

More formally, assuming two probability density functions $f_A (x)$ and $f_B (x)$, the Overlapping Index $\eta : \mathbb {R}^n \times \mathbb {R}^n \rightarrow [0,1]$ is defined in the following way:

1 $$\begin{aligned} \eta (A,B) = \int _{\mathbb {R}^n} min [f_A (x),f_B (x)] dx \end{aligned}$$

where, in the discrete case, the integral can be replaced by summation. As previously mentioned, η(A,B) is normalized to one and when the distributions of A and B do not have points in common, meaning that $f_A (x)$ and $f_B (x)$ are disjoint, η(A,B)=0. This index provides an intuitive way to quantify the agreement between *A* and *B* based on their density functions (Inman & Bradley, 1989).

To quickly illustrate an example of the overlapping area, Fig. 1 represents two different empirical densities. In panel [A], two density distributions are depicted, a Normal(10,2) and a Uniform(0,20); note that the two distributions have the same mean (10), but different variances, 4 and 33.3 respectively. In the panel [B] are represented the empirical densities of two random samples of 30 observations drawn from the two populations specified as in panel [A]; the estimated overlapping area being η=0.46.

Figure 1 shows how two distributions with almost the same mean could still be very different from each other with the overlapping area being $\hat{\eta } = 0.46$. In this case, the *t*-test focuses on mean differences, therefore correctly does not reject the null hypothesis, even though the degree of similarity of the two densities is only 46%, in other words, the difference is about 54%. Moreover, we remind readers that the *t*-test in this case is far from ideal as the two distributions have different variances.

Of note, although the $\zeta _{\textrm{ov}}$ test is nonparametric in that it does not assume any specific parametric form for the underlying distributions and relies on permutation for inference, it does require estimating empirical density functions using kernel density estimation (KDE). Like all nonparametric estimators, KDE introduces smoothing parameters (e.g., bandwidth) that can affect the precision of the overlap estimate. While standard bandwidth rules (e.g., Silverman’s rule) work well in many scenarios, special care is required in cases of bounded data, where standard kernels may yield biased estimates near the boundaries. In such cases, boundary-corrected KDE methods or data transformations (e.g., logit for proportion data) can be used to improve accuracy. We note that KDE is widely accepted in psychological research (e.g., for modeling reaction time distributions). Nonetheless, researchers should be mindful of KDE characteristics when applying the method in bounded or sparse data contexts.

### Permutation approach

Permutation tests, also known as randomization tests, are a class of nonparametric statistical significance tests. The concept dates back to the work of R.A. Fisher in the 1930s, in particular his book *The Design of Experiments* (Fisher, 1935). The theoretical foundations were further developed by E.J.G. Pitman in his seminal papers of 1937 (Pitman, 1937) and 1938 (Pitman, 1938). The basic principle of permutation testing is based on the idea of rearranging observed data to generate a null distribution. This approach assumes that if the null hypothesis is true, then all possible arrangements of the data are equally likely, i.e., each permuted sample has the same probability as the observed one. By resampling the data, we can obtain the distribution of the test statistic under the null hypothesis without making any assumptions about the underlying data generating process. This is particularly valuable when dealing with small sample sizes or when the assumptions of parametric tests are not met. The observed test statistic is then compared to this empirically derived null distribution to determine the probability of obtaining such a result by chance alone (Pesarin, 2001).

The permutation approach allows the adoption of any test statistic chosen by the user, including statistics designed for paired samples. In the case of paired data, observations are not exchanged between participants; instead, condition labels are permuted within each participant. This means that the relationship between paired observations is preserved while testing whether the observed difference between conditions could arise by chance. As a result, the procedure naturally accounts for individual differences while maintaining the logic of permutation-based inference. For example, if we are thinking about comparing the means of the two manipulation checks within a sample, we could choose a *t*-test statistic for repeated measures; the data in the two conditions are permuted and the *t* value is calculated each time. If the two conditions come from the same population, the *t*-statistic computed on the observed data should be close to 0; the *t*-statistic computed on randomly permuted data will also give values close to zero. Therefore, the randomly generated test statistic and the observed one have the same – nonparametric – distribution. Otherwise, if the two groups come from populations with different means, the *t*-statistic computed on the observed data will be far from zero, while the *t*-statistic computed on the permuted data will be around zero.

The *p*-value is the probability of obtaining an equally or more extreme *t*-statistic compared to the observed one:

2 $$\begin{aligned} p=\frac{(\#_{b=1}^B |t_b|\ge |t|)+1}{B+1} \end{aligned}$$

where *B* is the number of random permutations, *t* is the *t*-statistic computed on the observed data, $t_b$ are those computed on the permuted data.

The test will have power – i.e., the probability of getting a $p\le \alpha$ when the two conditions or two samples are really different – very close to the parametric *t*-statistic, and it will retain control over false positives even when the assumptions of normality are not met.

**Fig. 2:**
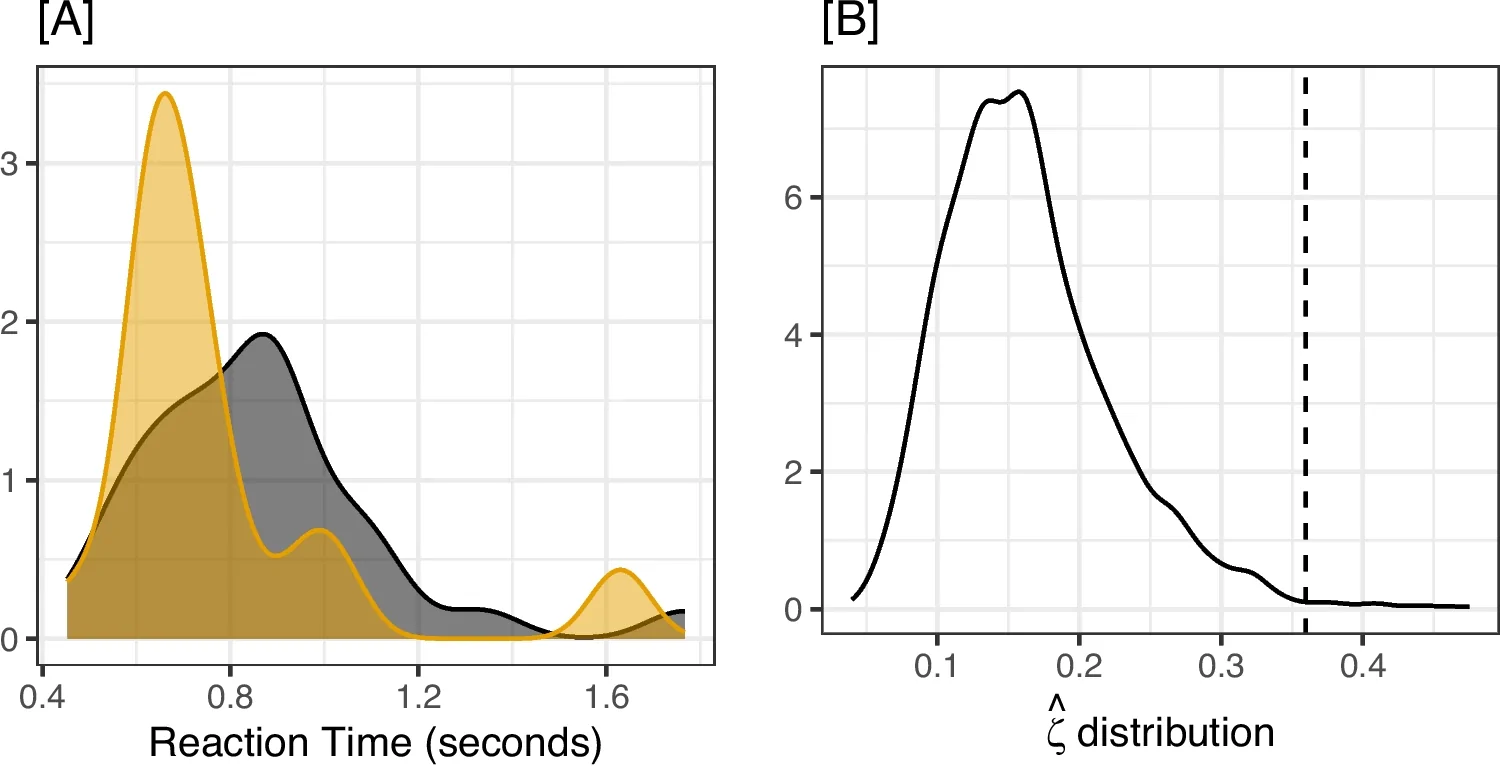
[**A**] Distribution of reaction times for high- and low-frequency words in English, plotted with a colour-blind friendly palette. The overlapping area is $\hat{\eta } = 0.64$, corresponding to a non-overlapping area of $\hat{\zeta } = 1 - \hat{\eta } = 0.36$; [**B**] Distribution of $\hat{\zeta }$ obtained with 3000 permutations of the data

It is important to note that the choice of which *t*-statistic to use is the user’s choice; different test statistics (e.g., difference of mean ranks, Kolmogorov-Smirnov, etc.) will produce tests with different power. For example, if the two samples differ only in their variability and not in their mean, the permutation test based on the *t*-statistic will have little or no power to detect that the two samples come from different populations. In this direction, the present paper proposes to use the Overlapping Index as a test statistic which results proves to be powerful under a wide range of differences in distributions.

#### Remark 1

The choice to add a +1 in the numerator and denominator is a choice supported by many authors (Hemerik & Goeman, 2018; Phipson & Smyth, 2010) and ensures that the probability of false positives is less than or equal to α.

#### Remark 2

As one can understand, the *p*-value may change depending on the permutations that are drawn. By increasing the number of permutations *B*, the results will change less and less. Since the number of possible permutations is finite, it is preferable, if possible, to explore the set of them (i.e., to compute the statistics on all possible permutations of the data). This set of all possible rearrangements of the data is, in fact, the orbit of the sample that allows us to compute the exact *p*-value - i.e., the exact probability of observing a test statistic that is as extreme or more extreme than that observed in the data. In this case, $$B=\left( {\begin{array}{c}n\\ n_1\end{array}}\right) = \frac{n!}{n_1!(n-n_1)!}$$ and the *p*-value formula reduces to

$$ p=\frac{(\#_{b=1}^B |t_b|\ge |t|)}{B} $$

since the test statistic computed on the observed data is certainly in this set.

### Application of permutation test to the overlapping index

Even though the Overlapping Index has a simple interpretation, it does not provide information about the statistical significance of η. Therefore, we implemented permutation testing to provide a measure of statistical significance. In particular, we implemented permutation testing to give a tool that tests differences in distributions without assumptions, offering a valid alternative in cases in which traditional assumptions are not met.

If we are reasoning from the perspective of Null Hypothesis Significance Testing (NHST), we should define the null hypothesis as follows: $H_0: \eta = 1$, meaning that there is complete overlap between the theoretical densities in the two populations from which we sample the data. For this reason, it is more intuitive to work with the complement of η, which is $1-\eta = \zeta$ which is the area of non-overlap, therefore, defining the null hypothesis as $H_0:\zeta = 0$, once again meaning that there is no difference between the densities of the two populations. Obviously, this does not change the results, but only the way in which they are interpreted. When testing the difference between the two distributions, we will no longer be working with η, but with the complement ζ.

The algorithm estimates the value of ζ on the observed data ($\hat{\zeta }$). Then, through permutation, the observed values of the two groups are randomly re-assigned to the groups for B times, estimating again the new value of $\hat{\zeta }_b$. The times in which the estimate of $\hat{\zeta }_b$ on permuted data is greater than or equal to the one observed on real data is estimated ($\hat{\zeta }_b \ge \hat{\zeta }$) and then the found value is divided by B, returning the *p*-value.

### Illustrative example with real data

To show a realistic application and possible workflow, we present a real case of a dataset available online (Oksuz & Rebuschat, 2024)^1^ on reaction times in word reading of high- and low-frequency words in English. For the applied example we selected two conditions of word reading of high and low frequency words in English from 29 participants.

#### Step 1: descriptive statistics and data visualization

The first step is to plot the two conditions/groups (Fig. 2, panel [A]). This can easily be done with the overlapping R package (Pastore et al., 2024). First, the user has to load the package into the R environment, then a list object is created with the two vectors of values of the two groups/conditions, subsequently the function overlap is used allowing users to easily plot densities by setting plot = T. Here is a quick illustration of code:

**Figure:**
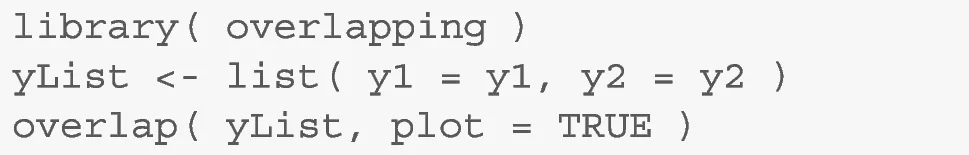

**Table 1:** Descriptive statistics for low and high frequency words

	Low frequency	High frequency
Mean	0.86	0.77
Median	0.87	0.69
Variance	0.07	0.07
Skewness	1.47	2.20
Kurtosis	6.25	7.49

Simultaneously, calculate descriptive statistics (see Table 1). The two conditions have means of 0.86 and 0.77, variance of 0.07 for both conditions, and skewness of 1.47 and 2.2, for low- and high-frequency respectively. The overlapping area between the two distributions is η=0.64, and consequently ζ=1-0.64=0.36. Already from this information, one can gain insight into the characteristics of the data.

#### Step 2: Significance testing

After visualizing the data and carefully evaluating descriptive statistics, one can move to hypothesis testing. When a global test is appropriate for the research question, or when sample size is small and assumptions are violated, a permutation test based on the Overlapping Index is ideal. To perform the $\zeta _{\textrm{ov}}$ test, the procedure is the same as for the overlap function, with the only difference that the function perm.test is used instead, as shown below:

**Figure:**
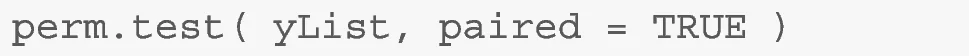

The function will give the following output:

**Figure:**
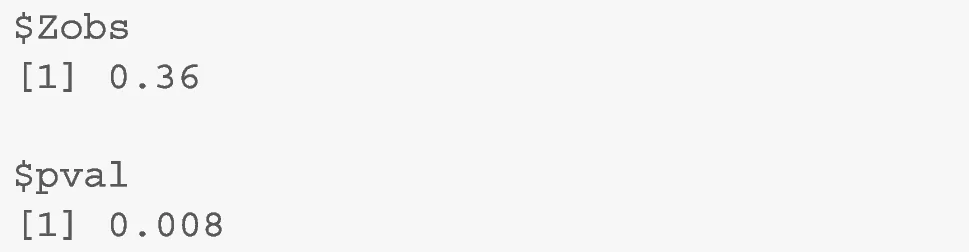

Zobs is the observed ζ and pval is the *p*-value obtained through permutations.

Figure 2[B] is represents the distribution of the values of $\hat{\zeta }$ obtained with 3000 permutations; the *p*-*value* is calculated as follows:

$$\begin{aligned} p = \frac{(\# \hat{\zeta }_b \ge \hat{\zeta })+1 }{B+1} = \frac{24}{ 3001 } \end{aligned}$$

Using permutations to obtain a *p*-value for ζ gives a p<.01. Based on this test, we can conclude that there is a statistically significant difference between the two distributions, with an area of non-overlap equal to 0.36. Instead, performing a *t*-test gives a non-significant result: t(28)=1.46, p=.16.

This result suggests that the overlapping method has detected differences that the *t*-tests did not identify, highlighting the potential sensitivity of this approach.

In our example, we believe that no additional testing is needed, as the graphical representation, the descriptive statistics and the $\zeta _{\textrm{ov}}$ test give a clear insight into the difference between the two conditions, with a ζ=0.36 and a clear delay in reaction time of the Low frequency condition.

Similarly, if two groups were not to differ significantly, especially with a small sample, the researcher should have focused on the effect size and reason whether the area of non-overlap could reasonably be considered a trivial difference (based on the field of study and the specific effect) or if more data was needed to reach a meaningful conclusion. One cannot simply reject $H_0$ and should carefully evaluate the effect size, as absence of significance does not mean absence of an effect. In this case, reasoning about the smallest effect size of interest (SESOI) and running a sensitivity analysis (ideally a priori power analysis should be run before the data collection) can be beneficial for the researcher.

This approach highlights the importance of visualizing data and stresses the invaluable insight offered by descriptive statistics (Pastore et al., 2017; Tay et al., 2016; Wilkinson, 1999). However, in case of clear differences araising in Step 1 and confirmed in Step 2, one can decide to move forward with Step 3.

#### Step 3: Tailored parameter specific testing

Optional for deeper inquiry, this phase encourages researchers to openly weigh theoretical hypotheses against data characteristics, selecting tools (such as independent t-tests/Welch for mean differences, Levene’s/F for variances, or skewness/kurtosis tests via Kolmogorov-Smirnov) in a critically informed way. Data quality and structure guide these choices, with clear labeling of confirmatory vs. exploratory elements to address type I error concerns transparently (cf. p-hacking simulations (Stefan & Schönbrodt, 2023) ). Such an open, layered approach helps unpack distributional differences captured by ζ, balancing rigor with adaptability.

We also believe that Step 2 can be replaced by Step 3 if the researcher has a clear parameter specific hypothesis and descriptive statistics support such a choice by not showing clear assumptions violations.

**Fig. 3:**
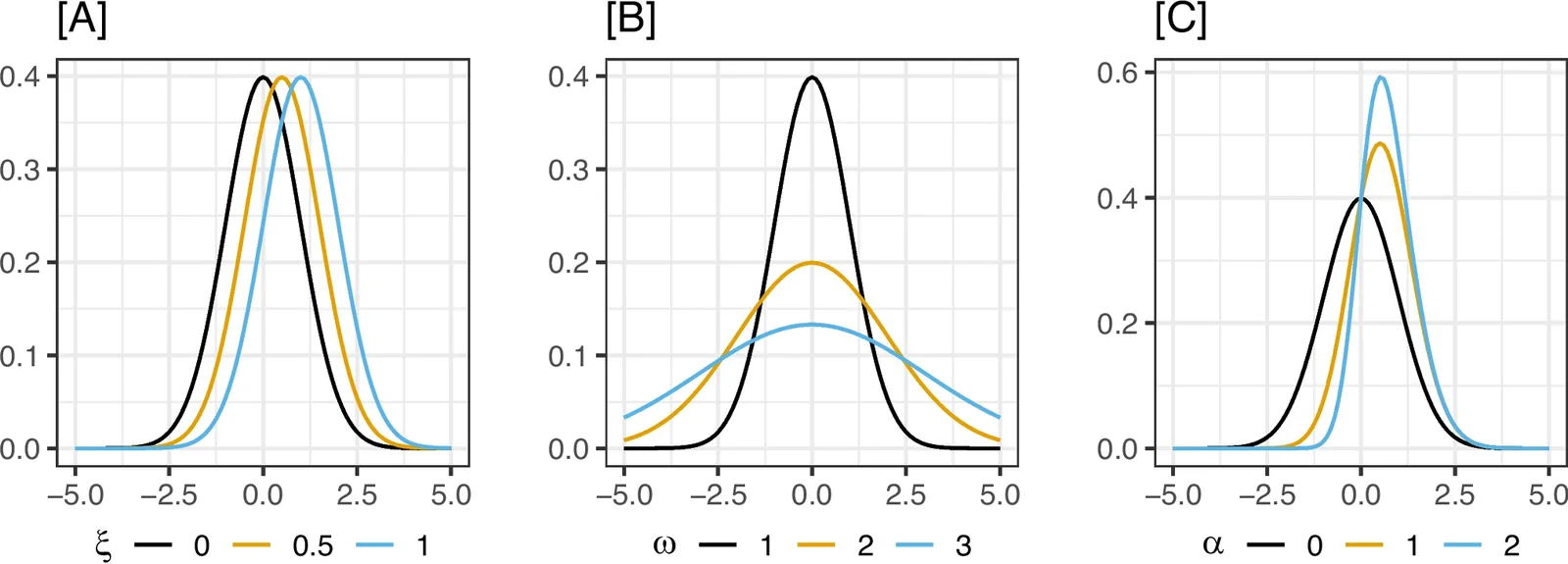
Examples of Skew-Normal distributions (ξ,ω,α); [A] three densities with same scale and shape but different location parameter values (ξ=0,0.5,1), [B] three densities with same location and shape but different scale parameter values (ω=1,2,3) and [C] three densities with same location and scale but different shape parameter values (α=0,1,2)

### The simulation study

To evaluate the performance of the permutation test applied to the Overlapping Index, we performed a simulation study. The aim is to generate data for a set of scenarios distinguishing mean, variance and shape of the populations and compare the ζ perm test to other commonly used tests in terms of type I error control and power.

### Data generation

In the simulation, two density distributions will be compared for many different scenarios. The first distribution will always be a normal standard distribution with μ=0 and σ=1. To simulate data for the second distribution we use the Skew-Normal distribution (Azzalini, 1985), which is defined in the following way: given $\xi \in \mathbb {R}$, $\omega \in \mathbb {R}^{+}$ and $\alpha \in \mathbb {R}$, then for $y \in \mathbb {R}$ we have

3 $$\begin{aligned} \mathcal{S}\mathcal{N}(y|\xi , \omega , \alpha ) \!=\! \frac{1}{\omega \sqrt{2\pi }} \exp \left[ -\frac{1}{2} \left( \frac{y-\xi }{\omega } \right) ^2 \right] \left[ 1 \!+\! \text {erf}\left( \alpha \left( \frac{y-\xi }{\omega \sqrt{2}}\right) \right) \right] \end{aligned}$$

in which

$$ \text {erf}(z) = \frac{2}{\sqrt{\pi }} \int _{0}^{z} e^{-t^2} dt $$

is the *error function*. When ξ=0, ω=1 and α=0 the distribution is a standard normal distribution.

ξ is the location parameter, ω is the scale parameter and α is related to the skewness of the distribution. Therefore, this distribution is suitable to generate data modelling the distance between means (the effect size), symmetry and variance.

Mean and variance of the Skew-Normal are respectively:

4 $$\begin{aligned} \begin{array}{l} \mu = \xi + \omega \gamma \sqrt{2/\pi } \\ \sigma ^2 = \omega ^2 [1- (2\gamma ^2)/\pi ] \end{array} \end{aligned}$$

in which $\gamma = \alpha / \sqrt{1 + \alpha ^2}$. Based on the Eq. 4 we can determine the values to assign to the parameters ξ and ω as function of μ and σ with the equations:

5 $$\begin{aligned} \begin{array}{l} \xi = \mu - \omega \gamma \sqrt{2/\pi } \\ \omega = \sqrt{\sigma ^2/ [1- (2\gamma ^2)/\pi ]} \end{array} \end{aligned}$$

The Skew-Normal distribution is optimal for our purpose as it allows control over parameters of mean, variance, skewness and kurtosis, as shown in Fig. 3.

### Simulation design

In the simulation we compare two samples extracted from a Skew-Normal, the first one is generated from $\mathcal{S}\mathcal{N}(0,1,0)$, which is the Standard-Normal distribution, and the second one from $\mathcal{S}\mathcal{N}(\xi ,\omega ,\alpha )$. Consequently, the first sample always derives from a population with mean 0 and variance 1. To define the various scenarios, we manipulate the parameters of the second population in other to obtain specific differences in means (δ), standard deviations (σ) and skewness (α). Four factors were systematically varied in a complete four-factor design as follows:

- δ=(0,0.2,0.5,0.8); mean of the second population, which also corresponds to the difference between the two groups, the first one always has μ=0;
- σ=(1,2,3); standard deviation of the second population;
- α=(0,2,10); degree of asymmetry (skewness) of the second population;
- n=(10,20,50,100,500); sample size, equal in the two samples.

For each of the $4 \times 3 \times 3 \times 5 = 180$ conditions we generated 3000 sets of data on which we performed the analysis.

**Fig. 4:**
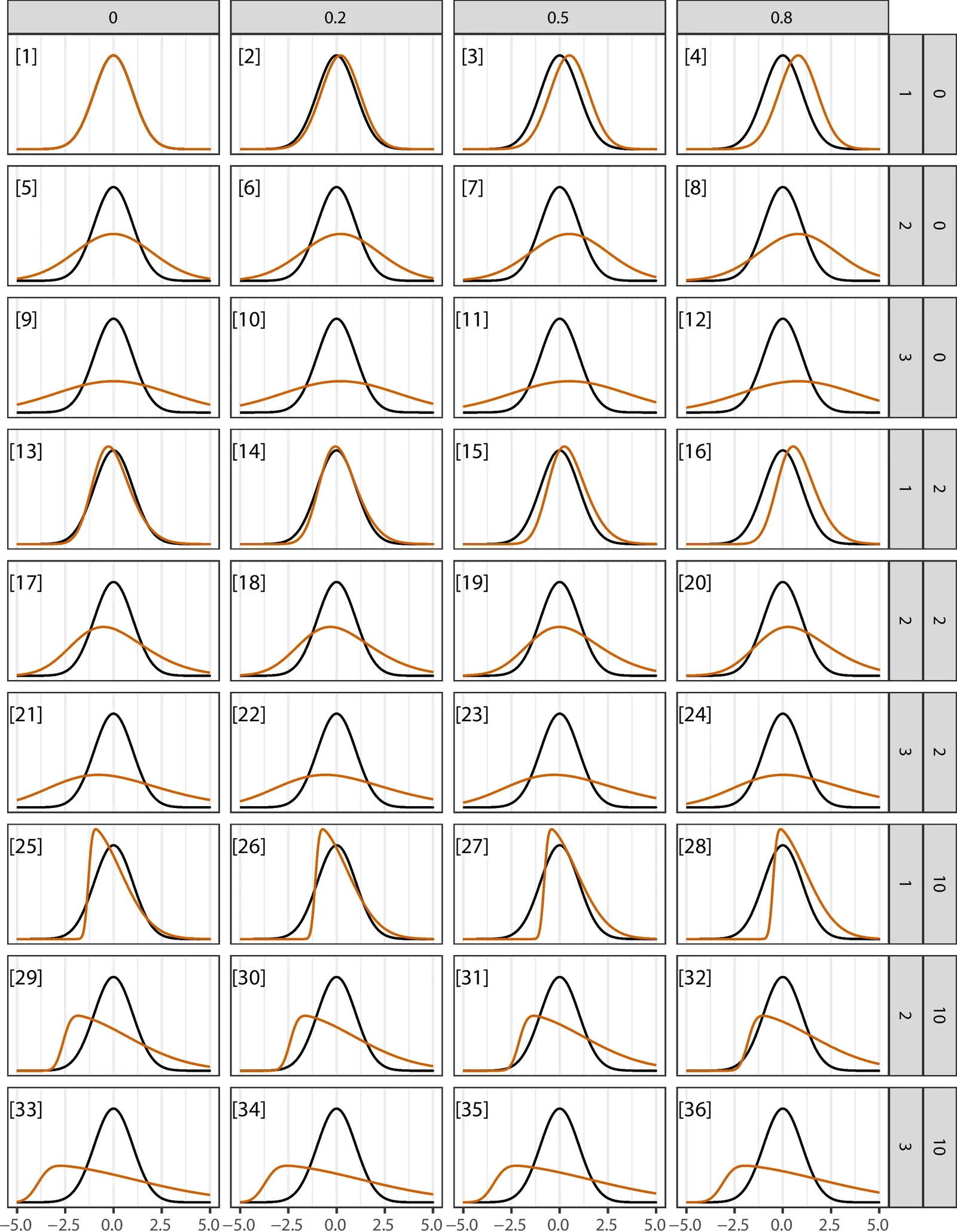
Generative data distributions in function of δ (column panels), σ and α (row panels). The black curves are the first population, $\mathcal{S}\mathcal{N}(0,1,0)$, and the orange curves represent the second population, $\mathcal{S}\mathcal{N}(\xi ,\omega ,\alpha )$

Figure 4 graphically represents the 36 scenarios of data generation, the black curves are the first population, always a $\mathcal{S}\mathcal{N}(0,1,0)$, and the orange curves refer to the second population $\mathcal{S}\mathcal{N}(\xi ,\omega ,\alpha )$.

For each combination $\delta \times \sigma \times \alpha \times n$, the following tests were performed on the generated data:

- *t* test for independent samples, assuming equal variance;
- Welch test for independent samples;
- Wilcoxon test for independent samples;
- Permutation test on the complement of the Overlapping Index, $\zeta = 1-\eta$, which therefore becomes an index of difference between groups;
- *F* test of homogeneity of variances;
- Kolmogorov-Smirnov test for comparing two distributions.

The whole procedure generated a total of 540000 datasets as well as 3240000 statistical tests and corresponding *p*-values.

### Definition of statistical tests

We introduce the chosen statistical tests summarizing the specific hypothesis and assumptions for each one.

#### *t* test

This is the classic case of a test for independent samples assuming equal variances and the normality of the two distributions:

$$\begin{aligned} H_0: \mu _1 - \mu _2 = 0 \text{ with } \sigma ^2_1 = \sigma ^2_2 \end{aligned}$$

Therefore, in the scenarios from which the samples come from populations with the same mean – Fig. 4, panels in the first left column, [1, 5, 9, 13, 17, 21, 25, 29, 33] – type I error control is estimated, whereas power is estimated for the remaining scenarios. Note that assumption of homogeneity of variance for this test is met only in the scenarios in the first row.

#### Welch (W) test

This is the *t* test modified when homogeneity of variances is not respected:

$$\begin{aligned} H_0: \mu _1 - \mu _2 = 0 \text{ with } \sigma ^2_1 \ne \sigma ^2_2 \end{aligned}$$

Also this test assumes normality.

Control of type I error is estimated for the same scenarios as for the *t* test, as well as for power.

#### Wilcoxon-Mann-Whitney (WMW) test

This is the test on ranks which evaluates the following hypothesis without assumptions on distributions:

$$\begin{aligned} H_0: P(X_1> X_2) = P(X_2 > X_1) = 0.5 \end{aligned}$$

in which $X_1$ and $X_2$ are the random variables representing the observations extracted from the two populations. In this case, the only scenario in which $H_0$ is true is in panel [1]. Given that this is a distribution-free test, assumptions are not required.

#### Kolmogorov-Smirnov (KS) test

This test compares the cumulative distributions

$$ H_0: F(X_1) = F(X_2) $$

without assumptions on distributions. The null hypothesis is true in panel [1], as it is for the ζ permutation test.

#### *F* test

This is the test of homogeneity of variances

$$ H_0: \sigma ^2_1 = \sigma ^2_2 $$

assuming normality. The condition is true in all scenarios where σ=1, panels [1-4, 13-16, 25-28]. In those scenarios we estimate type I error, in all the others we calculate power.

#### $\zeta _{\textrm{ov}}$ test

Since $\zeta = 1 - \eta$, in which η is the area of overlapping of the empirical distributions, the null hypothesis of the test is

$$ H_0: \zeta = 0 $$

which implies that the data come from the same population, or from populations with the same shape (mean, variance and skewness) but without specific assumptions. Therefore, the only condition in which $H_0$ is true is the first panel, [1]. Also in this case, the test does not require particular assumptions.

## Results

First of all, we analysed correlations between the *p*-values of the considered tests in order to assess how much they are associated independently from the experimental condition. Next, we considered separately for each test in which scenarios $H_0$ is true, as reported in the previous section. Consequently, we computed type I error by counting how many times the test is significant in those scenarios, and the power by counting how many times it is significant in all other scenarios. In this way, we evaluated type I error and power based on the experimental conditions.

### Correlations among tests

**Fig. 5:**
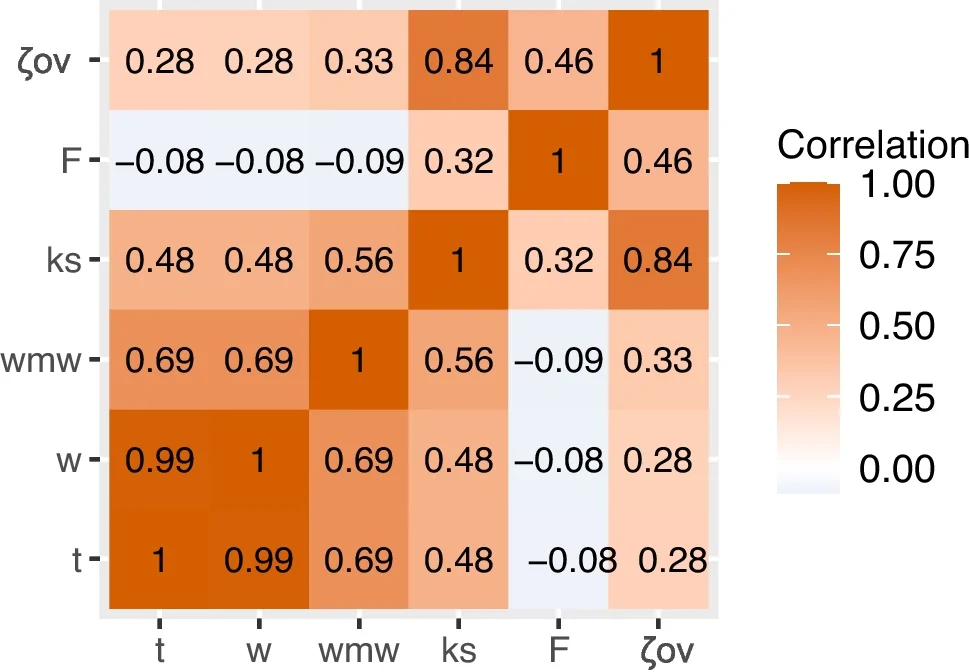
Correlation matrix among *p*-values (N=540000) in chosen tests. Note: $\zeta _{\text{ ov }}$ = ζ overlapping test, *F* = variance test, ks = Kolmogorov-Smirnov test, wmw = Wilcoxon-Mann-Whitney test, w = Welch test, *t* = Student’s *t* test

Figure 5 represents the correlation matrix between the *p*-values for the different tests in all experimental conditions. The classical tests show an order in the way they correlate. More specifically, *t* and W tests show a perfect correlation, WMW is highly correlated with the aforesaid tests, and the KS shows a lower but still medium-large correlation. *F* presents no correlation with *t*, W and WMW tests, and a medium correlation with the $\zeta _{\textrm{ov}}$ and KS tests.

The $\zeta _{\textrm{ov}}$ test is highly correlated with the KS test, has a lower correlation with tests on means (*t* and W) and ranks (WMW), and a medium correlation with the *F* test.

The lower correlations observed among the test statistics are expected, given that each test is designed to detect different aspects of the data - whether central tendency, variance, or overall distributional shape. This pattern reinforces the idea that tests are not interchangeable but rather complementary in what they capture. The $\zeta _{\textrm{ov}}$ test, by design, integrates sensitivity to multiple features, which explains its lower correlations with more narrowly focused tests. Therefore, while the correlation matrix does not offer direct interpretive insight into test performance, it supports the broader claim that distinct tests yield distinct inferential perspectives.

### Type I error and power

**Fig. 6:**
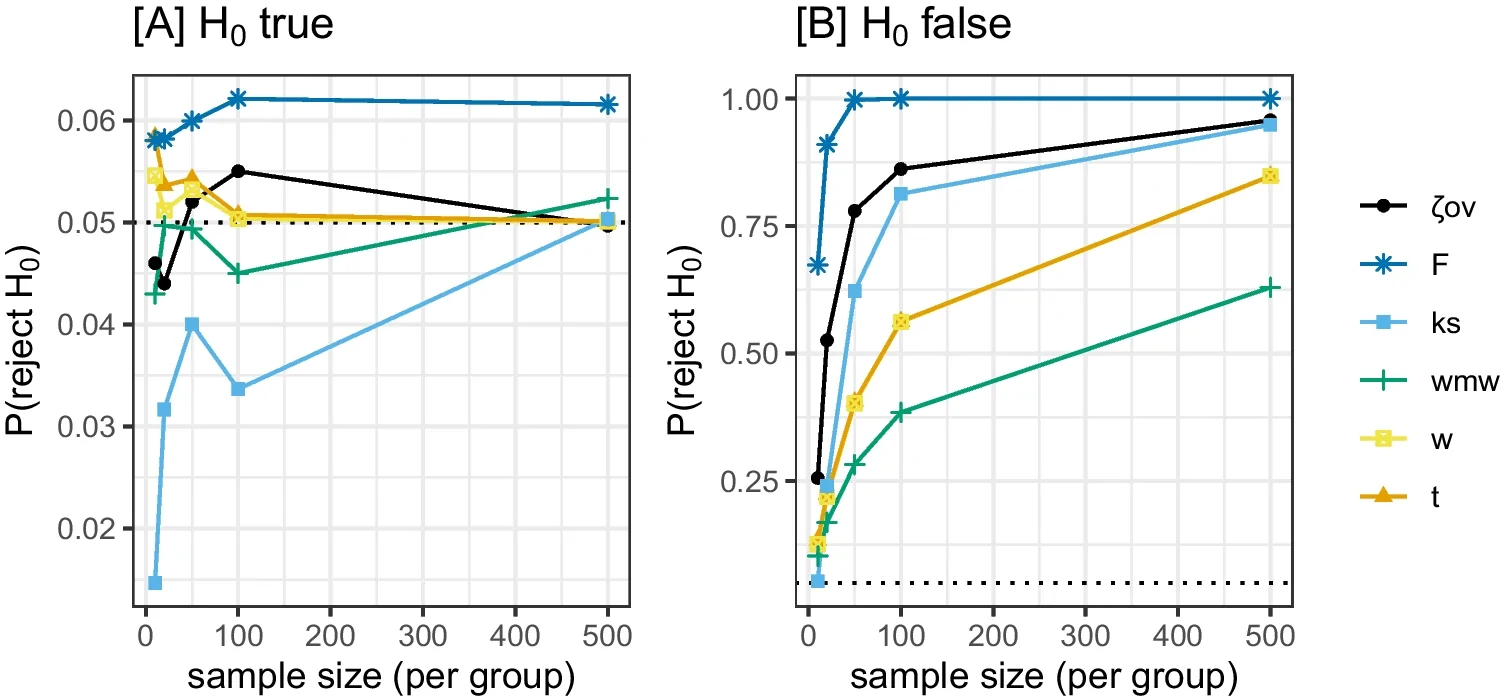
Control of type I error [**A**] and power [**B**] in the various tests. Note: $\zeta _{\textrm{ov}}$ = $\zeta _{\textrm{ov}}$ test, *F* = variance test, ks = Kolmogorov-Smirnov test, wmw = Wilcoxon-Mann-Whitney test, w = Welch test, *t* = Student’s *t* test

Figure 6 shows the type I error in panel [A] and power in panel [B], estimated considering the situations reported in the section *Definition of Statistical tests* as true null hypothesis.

#### Remark

It is important to note that the six tests compared in this simulation evaluate different null hypotheses and are therefore sensitive to different aspects of distributional differences. Specifically: the $\zeta _{\textrm{ov}}$ test evaluates $H_0: \zeta = 0$ (complete distributional overlap); the *F*-test evaluates $H_0: \sigma ^2_1 = \sigma ^2_2$ (homoscedasticity); the Kolmogorov-Smirnov test evaluates $H_0: F(X_1) = F(X_2)$ (identical cumulative distribution functions); the Wilcoxon-Mann-Whitney test evaluates $H_0: P(X_1 > X_2) = 0.5$ (stochastic equality); the Welch test evaluates $H_0: \mu _1 = \mu _2$ allowing unequal variances; and the Student’s *t*-test evaluates $H_0: \mu _1 = \mu _2$ assuming equal variances. This aspect is taken into account when defining for which scenarios to compute power and when to compute Type I error, ensuring a fair comparison between tests.

In relation to type I error, almost all tests show a good performance, whereas the KS test is too conservative for small samples and the *F*-test is always above the nominal level of 0.05 (even with 500 observations per group).

Concerning power, the $\zeta _{\textrm{ov}}$ test is the second-best-performing test, outperforming other tests with good control of Type I error, already with small sample sizes, with the only exception of the *F*-test, which has higher power but bad control of Type I error. From the graphical representation it is visible how two subgroups can be identified: one including the tests on means and ranks, not reaching adequate power even with large samples, and the second one formed by the $\zeta _{\textrm{ov}}$ and KS tests, reaching good power already from 100 observations, with the $\zeta _{\textrm{ov}}$ outperforming the KS test reaching good power already from 50 observations.

## Discussion

The analysis of *p*-value correlations among the tests provides insight into their relationships. High correlation between the *t*-test and W test, for instance, reflects their similar objectives and shared focus on mean differences. However, lower correlations between parametric and non-parametric methods, such as the WMW and KS tests, indicate that these tests capture distinct aspects of the data, such as ranks or distribution shapes rather than means. The permutation-based tests show intermediate correlations with both parametric and non-parametric methods, which suggests that they may align with either type of test depending on the underlying data structure, highlighting the versatility of the $\zeta _{\textrm{ov}}$ test.

Moreover, the present analysis evaluated the behavior of several significance tests across simulated scenarios, while explicitly acknowledging that the tests do not all target the same null hypothesis. The parametric tests (*t*-test, Welch test, and *F*-test for variance), non-parametric tests (Wilcoxon–Mann–Whitney and Kolmogorov–Smirnov), and the permutation-based $\zeta _{\textrm{ov}}$ test answer related but distinct inferential questions. Consequently, differences in Type I error control and power should be interpreted in relation to each test’s target null hypothesis rather than as a direct hierarchy of performance.

In Fig. 6, panel [A], the only test which is over-conservative in terms of Type I error is the KS test, yet reaching the nominal .05 level with bigger samples (over 400 observations). Instead, the *F*-test shows a bad control of Type I error, always exceeding the nominal level even with large sample size. All other tests, even from small samples, are in the range of .045-.055, converging closer to .05 as sample size grows. From this analysis we can then conclude that most of the tests sufficiently control the Type I error, but caution is needed for the *F*-test even with big samples and with the KS test with small samples.

Concerning sensitivity, Fig. 6 panel [B] shows that the *F*-test is highly sensitive to variance differences, although this result should be interpreted alongside its Type I error behavior. The $\zeta _{\textrm{ov}}$ test also shows high sensitivity to global distributional differences in the simulated scenarios. The KS test shows comparable sensitivity in larger samples. The W and *t* tests show nearly identical behavior, consistent with their almost perfect correlation of *p*-values, and are less sensitive in scenarios where the difference is not limited to central tendency. Lastly, the WMW test performs similarly to *t* and W tests with slightly less power as sample size increases. These results suggest that the $\zeta _{\textrm{ov}}$ test is sensitive to distributional differences and its use should be preferred when the research question concerns global distributional differences.

While the $\zeta _{\textrm{ov}}$ test demonstrates consistently high power across the simulation scenarios, it is not designed to replace traditional tests focused on specific parameters like the mean. Rather, it offers a global option when researchers are not interested in a single parameter, or when data violate the assumptions of parametric tests. In this way, it complements rather than competes with existing approaches, and supports the growing shift in psychological science toward robust, distribution-aware inference.

Moreover, it is important to note that this performance reflects its broader sensitivity to differences in distributional shape, not just central tendency; therefore, it may detect effects that more narrowly focused tests (e.g., t-test or F-test) are not designed to identify. This feature is highlighted in the example presented in section “Application of permutation test to the overlapping index”, showing how the $\zeta _{\textrm{ov}}$ test is responsive to a wider set of deviations from the null.

The main limitation of the study is that it simulates only scenarios in which the first population is a Standard-Normal distribution ($\mathcal{S}\mathcal{N}(0,1,0)$) and it does not consider the presence of outliers, which would give more insight into the performance of the $\zeta _{\textrm{ov}}$ test. Another concern could be that the test does not provide information on specific parametric differences, as its design focuses on distributional overlap rather than mean differences, which means it does not directly inform on mean, variance or skewness differences specifically. But we argue that this is rather a core characteristic of the test and not a limitation. The $\zeta _{\textrm{ov}}$ test offers a useful starting point for evaluating whether two distributions differ at a global level, especially when the research question is not restricted to a single parameter. Moreover, the overlapping R package which easily computes the index, also offers the possibility to plot densities and the area of overlap, therefore making it extremely intuitive to visualize how the two distributions practically differ.

While the $\zeta _{\textrm{ov}}$ test offers a broader diagnostic scope, it is not intended to replace targeted hypothesis tests when those are clearly aligned with the research goal and respect the nature of the data. When the research hypothesis specifically targets a single parameter, such as a mean difference, the use of more focused tests (e.g., Welch’s t-test) may offer higher statistical power under valid assumptions. A complementary simulation study comparing power across such focused and omnibus approaches would be a valuable avenue for future work.

## Conclusion

Many researchers focus on differences in means and may not initially consider the full distribution of their data. One of the strengths of the $\zeta _{\textrm{ov}}$ test is precisely that it encourages a more holistic view, prompting researchers to explore whether groups differ not just in central tendency but in dispersion or shape as well. More precisely, it is easy to interpret as an effect size, with high values of ζ signaling differences between the two empirical distributions and low values indicating similarity. It is robust to distributional assumptions, as it calculates *p*-values through permutations rather than relying on parametric assumptions like normality or equal variance, making it particularly useful in scenarios where other tests may be sub-optimal due to assumption violations, and, in the simulated settings considered here, showing adequate Type I error control and good sensitivity to global distributional departures. In practice, the $\zeta _{\textrm{ov}}$ test can serve as a global test that prompts a broader examination of the data’s characteristics.

By exploring alternative scenarios, the study offers a practical indication to operate a shift in the philosophical approach to data analysis and significance testing. In fact, the Overlapping Index forces the functional interpretation of the results to move beyond significance testing alone (Steegen et al., 2016; Gelman, 2018; Pastore & Calcagnì, 2019). In psychological research, considering the distribution of data rather than relying solely on significance testing offers a deeper, more nuanced understanding of results. Traditional significance testing does not provide information about the nature or magnitude of that effect (see Cohen, 1994; Wagenmakers, 2007; Ziliak & McCloskey, 2008; Wasserstein & Lazar, 2016). By visualizing and considering the entire distribution of data, researchers can observe the spread, central tendency, and shape of the data, which often reveal valuable insight about variability and individual differences within the sample. As in the example presented in Fig. 2, reporting a mean difference without an understanding of the data distribution could lead to misrepresentation of the consistency or generalizability of the observed effect. Therefore, incorporating distributional analyses allows psychologists to present a fuller picture of their findings, improving both interpretability and transparency in their research conclusions.

While classical concerns regarding normality and homoscedasticity tend to diminish with increasing sample sizes, the $\zeta _{\textrm{ov}}$ test offers unique advantages that persist even in large-sample scenarios. Specifically, it provides a formal and assumption-free way to test whether full empirical distributions differ beyond just location parameters, allowing researchers to assess global differences with one test. The permutation-based *p*-value offers a rigorous statistical complement to data visualization: while plotting distributions is essential, ζ offers an objective inferential check that enhances transparency and reproducibility, particularly when interpretation may be ambiguous.

Importantly, not all issues resolve with large sample sizes. Psychological measures such as reaction times and event-related potential (ERP) components typically exhibit non-normal, right-skewed distributions (Blanca et al., 2013; Luck & Gaspelin, 2017). Reaction-time modeling relies heavily on ex-Gaussian distributions (e.g., (Lacouture & Cousineau, 2008) because skew violates parametric test assumptions even with n>50. Meta-analyses show that only ∼ 5.5 % of behavioral datasets approximate normality (skew ≈ 0; kurtosis ≈ 0), even in moderate sample sizes (n 1030). These issues are compounded in physiological measures like ERPs, which often follow heavy-tailed distributions due to individual differences and noise. In these contexts, the Overlapping Index η and the $\zeta _{\textrm{ov}}$ test offer a robust, nonparametric alternative: it quantifies full distributional differences and provides *p*-values via permutation, without requiring normality or equal variances. Moreover, beyond traditional significance testing, the $\zeta _{\textrm{ov}}$ framework can be readily adapted for equivalence testing and minimum-effect testing (Lakens et al., 2018), in line with current recommendations (Riesthuis, 2024) Murphy & Myors, 1999). Because η (and its complement ζ) directly quantify the similarity or difference between empirical distributions, researchers can define meaningful thresholds (e.g., $\eta \ge 0.90$ or $\zeta \le 0.10$) that reflect negligible differences for practical purposes. A permutation-based test of equivalence can then assess whether the observed ζ falls within the predefined bounds, supporting equivalence. Conversely, a minimum-effect test can assess whether ζ significantly exceeds a lower threshold, indicating a difference of at least a meaningful size. Most importantly, thresholds should always be case specific and reasoned about, rather than conventional benchmarks. These extensions preserve the nonparametric and assumption-light nature of the $\zeta _{\textrm{ov}}$ test while allowing for more nuanced and informative inferential conclusions.

Moreover, the present study further underscores the necessity of reasoning about the most suitable statistical tools contingent on the specific characteristics of the data and the assumptions inherent in the analytical techniques employed. Such a switch in the philosophical approach to data analysis in the psychological sciences (Vasishth & Gelman, 2021) may improve the robustness and validity of psychological research findings, allowing for more informed interpretations and generalizations. We stress this by making data and material openly available so that such an approach might be useful for a wide range of psychologists interested in increasing the interpretability of their results.

Ultimately, statistics in psychology should reflect both theoretical knowledge and an appreciation for the distributional nuances of psychological variables. Rather than a rigid application of conventional methods, statistical analysis should be a deliberate choice that aligns with the nature of the data and the research question. The $\zeta _{\textrm{ov}}$ test embodies this principle, capturing the depth and complexity of psychological effects in a way that is both methodologically rigorous and sensitive to the real-world structure of psychological phenomena.

### Legenda

η is the area of overlap

ζ is the area of non-overlap, therefore 1-η

μ is the parameter of the mean of the normal standard

σ is the standard deviation of the normal standard

δ is the difference between the two means

ξ is the location parameter of the skew-normal

ω is the scale parameter of the skew-normal

α is the shape parameter of the skew-normal

## Data Availability

Data and materials to reproduce the present work are openly available at OSF

## Footnotes

1 Link to the dataset: https://osf.io/muwjz/files/ubtyq

## Code availability

The code to reproduce the present work is openly available at OSF
